# Effect of the Molecular Structure of TPU on the Cellular Structure of Nanocellular Polymers Based on PMMA/TPU Blends

**DOI:** 10.3390/polym13183055

**Published:** 2021-09-10

**Authors:** Ismael Sánchez-Calderón, Victoria Bernardo, Mercedes Santiago-Calvo, Haneen Naji, Alberto Saiani, Miguel Ángel Rodríguez-Pérez

**Affiliations:** 1 Cellular Materials Laboratory (CellMat), Condensed Matter Physics Department, University of Valladolid, Campus Miguel Delibes, Paseo de Belén n°7, 47011 Valladolid, Spain; mercesc@fmc.uva.es (M.S.-C.); marrod@fmc.uva.es (M.Á.R.-P.); 2 CellMat Technologies S.L., Paseo de Belén 9-A, 47011 Valladolid, Spain; v.bernardo@cellmattechnologies.com; 3 School of Materials, The University of Manchester, Oxford Road, Manchester M13 9PL, UK; haneen.naji@postgrad.manchester.ac.uk (H.N.); a.saiani@manchester.ac.uk (A.S.); 4 BioEcoUVA Research Institute on Bioeconomy, University of Valladolid, 47011 Valladolid, Spain

**Keywords:** nanocellular polymer, poly(methyl-methacrylate), thermoplastic polyurethane, gas dissolution foaming

## Abstract

In this work, the effects of thermoplastic polyurethane (TPU) chemistry and concentration on the cellular structure of nanocellular polymers based on poly(methyl-methacrylate) (PMMA) are presented. Three grades of TPU with different fractions of hard segments (HS) (60%, 70%, and 80%) have been synthesized by the prepolymer method. Nanocellular polymers based on PMMA have been produced by gas dissolution foaming using TPU as a nucleating agent in different contents (0.5 wt%, 2 wt%, and 5 wt%). TPU characterization shows that as the content of HS increases, the density, hardness, and molecular weight of the TPU are higher. PMMA/TPU cellular materials show a gradient cell size distribution from the edge of the sample towards the nanocellular core. In the core region, the addition of TPU has a strong nucleating effect in PMMA. Core structure depends on the HS content and the TPU content. As the HS or TPU content increases, the cell nucleation density increases, and the cell size is reduced. Then, the use of TPUs with different characteristics allows controlling the cellular structure. Nanocellular polymers have been obtained with a core relative density between 0.15 and 0.20 and cell sizes between 220 and 640 nm.

## 1. Introduction

Nowadays, modern society needs specific materials for each application, so there is a need to develop new and advanced materials as technology evolves. In this framework, a new generation of cellular polymers with enhanced properties was developed during the last decade: the so-called nanocellular polymers [1].

Nanocellular polymers are porous materials characterized by cell sizes in the nanoscale. These materials have aroused great attention owing to their very interesting combination of properties. As a result of their nanometric cell size, nanocellular materials present a reduced thermal conductivity of the gas phase due to the Knudsen effect [2,3,4]. Moreover, enhanced mechanical performance has been demonstrated in nanocellular polymers [5,6,7]. Furthermore, it is possible to produce semi-transparent nanocellular polymers [8,9], among other interesting properties [10,11].

The fabrication of nanocellular polymers is still a challenge, especially in the low-density range, because it requires a specific production technique to produce and stabilize cells in the nanoscale [1]. Among the diverse technologies employed for this purpose [3,12], one of the most promising methods is the so-called gas dissolution foaming process [13], which allows the bulk part production with a significant density reduction. To create a nanocellular structure, it is mandatory to promote high nucleation rates. High demanding process conditions such as high pressures or low saturation temperatures are needed to generate a nanocellular structure in pure polymers [14,15]. One interesting strategy to achieve high nucleation rates at mild processing conditions [16] is using nucleating agents to enhance nucleation. The nucleating agent increases the nucleation density to the values required to reduce the cell size to the nanoscale (higher than 10^13^ nuclei/cm^3^), allowing to produce nanocellular polymers. In the last years, several studies were conducted focusing on the effect of nucleating species in the fabrication of nanocellular polymers, such as nanoparticles [17,18,19,20] or an immiscible polymeric second phase [21,22]. For instance, Liu et al. [23] synthesize silica nanoparticles with different surface roughness to enhance the nucleation of poly(methyl-methacrylate) PMMA. They employed the same quantity of silica nanoparticles on each compound (around 10^13^ particles/cm^3^) and observed that increasing the surface roughness promotes a higher nucleation density because the energy barrier which promotes the cell nucleation is lower for concave surfaces than for flat-convex surfaces [24]. Yeh et al. [25] studied nanocellular thermoplastic polyurethane (TPU) polymers by adding graphene nanoparticles or adjusting the chemical structure of the TPU, obtaining cell sizes around 700 nm and relative densities between 0.8–0.9. They showed that adding nanoparticles as nucleation agents were more effective than increasing the hard segment (HS) content or replacing the soft segments to obtain homogeneous nanocellular structures and increase the nucleation density. In the work of Ni et al. [26], nanocellular polylactic acid (PLA) was produced by regulating the polymer crystals’ shape and degree of perfection. In this case, the crystals were proved to act as nucleating points. As the crystals become imperfect and with rougher surfaces, the nucleation density increases to 10^15^ nuclei/cm^3^, and the cell size decreases below 40 nm. Bernardo et al. [27,28] employed a block copolymer (poly(methyl methacrylate)-poly(butyl acrylate)-poly(methyl methacrylate) (MAM)) as a nucleating agent on a PMMA matrix. At high MAM concentrations, the copolymer forms spherical micelles that act as nucleating points [27]. However, as MAM content reduces up to 0.1 wt%, the micelles are not observed; however, the dispersed MAM molecules with high CO_2_ affinity can still increase nucleation [28]. The addition of only 0.1 wt% of MAM allows to increase the cell nucleation density three orders of magnitude and reduce the cell size five times, reaching 1·10^14^ nuclei/cm^3^ and cell sizes around 300 nm. Wang et al. [12] produced a polypropylene (PP) nanocomposite foam (cell size of 150 nm) using polytetrafluoroethylene (PTFE) nanofibrils as a nucleating agent. The nanofibrils increase the cell nucleation density up to five orders of magnitude and reduce the cell size 200 times compared to the neat PP foam.

Another interesting system to produce nanocellular polymers is the blend of PMMA with TPU. These two polymers are immiscible, and as a result, the TPU phase forms domains in the PMMA matrix. The TPU phase can lead to nanometric domains that can act as nucleation points with appropriate blending conditions. Wang et al. [29] produced a nanocellular polymer based on a blend of PMMA with 1 wt% of a commercial TPU with a minimum relative density of 0.125 and a cell size of 205 nm. These materials present a transition region between the nanocellular core and the external solid skin. The density was measured after removing the skin, but they do not specify if the transition region is removed or not. This is one of the best results reported in the literature regarding low-density combined with nanoscale cell sizes. However, they claimed that an additional cooling step before releasing the pressure was necessary to ensure a more homogeneous cellular structure. In our previous work [30], we use 2 wt% of a commercial TPU polymer to blend with PMMA to obtain graded cellular polymers with core relative densities between 0.16–0.30 and cell sizes ranging 310–480 nm on the nanocellular core. A graded cellular structure is characterized by variable cell size along the sample radius (samples showed a cylindrical shape). Thus, nanocellular PMMA/TPU samples are characterized by a nanocellular region in the core that gradually becomes microcellular toward the edge. We showed that the heterogeneous nucleation in the TPU domains is not predominant at all pressures [30]. Therefore, we hypothesized that the formation of the gradient of cell sizes is due to gradient nucleation in the PMMA/TPU samples, caused by a non-constant gas concentration profile in the samples due to the desorption of gas before the foaming step. Cellular materials with a gradient cellular structure (i.e., materials with regions of different cell sizes along the structure) can confer superior properties and provide the basis for developing new functions [31,32]. For this reason, several attempts to produce such structures have been developed recently, such as those of Pinto et al. [33] and Trofa et al. [34]. 

The previous papers dealing with nanocellular polymers based on PMMA/TPU blends employ commercial TPUs whose molecular structure is unknown (i.e., the type of the hard and soft segments and the hard segment content are not discussed in those papers). Thus, it was not possible to evaluate the effect of the TPU molecular structure on the resulting nanocellular polymer.

Based on the promising results obtained in the production of nanocellular polymers with the PMMA/TPU system, in this work, three specific TPU grades have been synthesized to be used as a nucleating agent for producing nanometric cells in PMMA. Three TPU grades with an amount of 60%, 70%, and 80% of HS were produced. The effect of the amount of HS in the TPU and of the TPU content (contents from 0.5 to 5 wt%) on the density and cellular structure of PMMA/TPU nanocellular polymers is investigated.

## 2. Materials and Methods

### 2.1. Materials

PMMA Plexiglas^®^ 7N was kindly supplied by EVONIK in the form of pellets. This PMMA presents a melt flow index (*MFI*) of 3.58 g/10 min (measured at 230 °C and 2.16 kg), a density (*ρ*) of 1.19 g/cm^3^, a glass transition temperature (*T_g_*) of 108.4 °C, measured by DSC (model DSC3+, Mettler). The molecular weight of this PMMA is *M_n_* = 45,000 g/mol and *M_w_* = 84,000 g/mol as determined by GPC.

The TPU grades under study were synthesized at the Polymers & Peptides Research Group (University of Manchester, Manchester, UK). Three TPU polymers with variable HS content were used in this study (60%, 70%, and 80% of HS content).

The TPU grades were synthesized with the following chemical compounds: 4,4-diphenylmethane-diisocyanate (MDI), poly(ethylene-glycol)-block-poly(propyleneglycol-block-poly(ethylene-glycol) (PEG-PPG-PEG) as macrodiol, 1,5 pentanediol (1,5-PDO) as chain extender, 1,4-Diazabicyclo[2.2.2]octane (DABCO) as catalyst and N,N-Dimethylacetamide (DMAc) as solvent. MDI in solid form has a molecular weight of 250.25 g/mol, a functionality of 2, a purity of 98%, a density of 1.18 g/cm^3^ at 25 °C, and a melting point of 42–45 °C. PEG-PPG-PEG, which forms the soft segments, has a number average molecular weight (Mn) of ~2000 g/mol, a functionality of 2, and a hydroxyl index of 56–59 mg KOH/g. The 1,5-PDO, which forms together with MDI the HS, has a molecular weight of 104.15 g/mol, a functionality of 2, and a purity of 97%. DABCO in solid form has a density of 1.02 g/cm^3^ at 25 °C and a melting point of 156–159 °C. DMAc has an average molecular weight of 87.12 g/mol and a melting point of −20 °C. All of them were supplied by Sigma Aldrich and, in general, used as received. PEG-PPG-PEG and 1,5-PDO were dried in a vacuum oven overnight at 80 °C and then were stored in sealed glass jars with molecular sieves to remove moisture. DABCO was deoxygenated by bubbling nitrogen before use.

Finally, medical grade carbon dioxide (CO_2_) (99.9% purity) was used as the blowing agent for the gas dissolution foaming experiments.

### 2.2. Synthesis of TPU Grades

**Figure 1:**
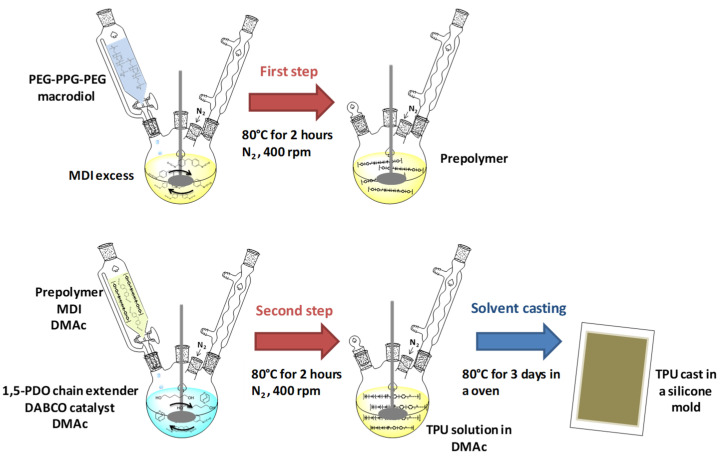
Scheme of TPU synthesis by prepolymer method.

Three TPU polymers were synthesized with different weight fractions of HS: 60% HS, 70% HS, and 80% HS (% indicated is by weight). TPUs were synthesized in a reaction flask by a two-step, prepolymer method under a dry nitrogen atmosphere (Figure 1). In the first step, PEG-PPG-PEG macrodiol was added dropwise from a funnel to an excess of MDI. This mixture was heated in an oil bath at 80 °C for 2 h with stirring at 400 rpm. In this step, it was obtained a prepolymer that contains a polyol end-capped by MDI. The prepolymer stoichiometric formation was calculated according to a molar ratio of the numbers of moles of the MDI and PEG-PPG-PEG polyol as 6:1 to cap all of the hydroxyl macrodiol groups with isocyanate groups of MDI. In the second step, the mixture of an additional amount of MDI and the prepolymer previously obtained in DMAc (240 mL) was added dropwise from the funnel to a preheated mixture of 1,5-PDO and DABCO (0.3 g, 0.3% with respect to the total mass of TPU obtained that is 100 g) in DMAc (60 mL). This reaction mixture was stirred at 400 rpm for 2 h in an oil bath at 80 °C. Finally, the solution containing the TPU was poured into silicone molds and maintained in an oven at 80 °C for three days to obtain TPU casts.

The amount of each component used for the different TPUs is collected in Table 1. The molar ratio of NCO/OH was adjusted as 1.02. In each synthesis, 100 g of TPU grades were produced.

### 2.3. Solid Blends Production

PMMA/TPU blends with different TPU contents were compounded using a twin-screw extruder model COLLIN TEACH-LINE ZK 25T, with L/D of 24 and screw diameter of 25 mm. Before compounding, PMMA and TPU were dried at vacuum at 60 °C for 12 h. Then, blends with the appropriate proportions were extruded using a temperature profile from 155 °C to 195 °C (in the die), increasing in intervals of 10 °C, and a screw speed of 40 rpm. The produced blends were cooled in a water bath and pelletized. After drying the pellets, the materials were extruded again under the same conditions to mix the two components homogeneously. In the second extrusion cycle, the extruded filament (average diameter between 3–4 mm) was set aside and let cool at room temperature. Blends with three TPU contents (0.5 wt%, 2 wt%, and 5 wt%) of the three TPU grades (60%HS, 70%HS, 80%HS) were produced. From now on, blends are named according to the amount and type of TPU. For instance, 0.5_60HS is the blend with 0.5 wt% of the TPU 60% HS. The cylindrical filament (that presents an average diameter of 3–4 mm) was cut in samples of 30 mm in length for the foaming experiments. Neat PMMA was processed under the same conditions for the sake of comparison. Figure S1 (Supplementary Information) shows a qualitative analysis of the solid samples produced in this work.

### 2.4. Gas Dissolution Foaming Experiments

Foaming experiments were performed in a high-pressure vessel (model PARR 4681) provided by Parr Instruments Company with a capacity of 1 L, capable of operating at a maximum temperature of 350 °C and a maximum pressure of 41 MPa. An accurate pressure pump controller (model SFT-10) provided by Supercritical Fluid Technologies Inc. automatically controls the pressure to keep the desired value. The vessel is equipped with a clamp heater of 1200 W, and its temperature is controlled via a CAL 3300 temperature controller. With this setup, foaming experiments were performed using a two-step gas dissolution foaming process [35]. Samples were firstly introduced in the pressure vessel under a CO_2_ pressure of 15 MPa and at 25 °C for the saturation stage. The saturation time was 20 h; this time is enough to ensure that the PMMA samples are fully saturated [36]. After saturation, the pressure was abruptly released using a pressure drop rate of 36 MPa/s during the first instants of the pressure drop. Pressure is released through an electro-valve to maximize pressure drop rate (key parameter to maximize nucleation in gas dissolution foaming [37]). Then, samples were removed from the pressure vessel and immersed in a thermal bath at the desired foaming temperature for the foaming step. Foaming was carried out in a water bath at 90 °C for 1 min. Foaming conditions were selected to maximize expansion without degeneration of the structure [30]. The time between the release of pressure and the immersion of samples in the bath was 2 min (time needed to fully release the pressure and open the vessel).

Note that 25 °C and 15 MPa are mild saturation conditions where the CO_2_ is in the compressive liquid state, helping to increase the amount of gas absorbed by the PMMA and promote a faster diffusion. Under these saturation conditions, the effective glass transition temperature of PMMA after the gas absorption is below room temperature [36], so samples start to expand immediately after the release of pressure. Nevertheless, this expansion is clearly smaller than the one that takes place when the samples are introduced in the thermal bath at 90 °C.

### 2.5. Characterization of TPU Grades

The TPU casts used to prepare the PMMA/TPU blends were characterized by density measurements, GPC and DSC. Moreover, compression-molded samples were required to characterize the shore hardness of TPU grades. The compression-molded samples were obtained by using a hot plate press. First, the TPU was heated in a mold of 2 mm of thickness at a temperature above melting temperature for 3 min, raising the pressure to 10 MPa at 0.5 bar/s. After that, the materials were pressed under the constant pressure of 10 MPa for 7 min, lowering the temperature to 60 °C at 25 °C/min.

#### 2.5.1. Density

Density of the TPU grades (*ρ*) was measured through a gas pycnometer (model AccuPyc II 1340, Micromeritics, Norcross, GA, USA).

#### 2.5.2. Gel Permeation Chromatography

Gel permeation chromatography (GPC) was used to obtain number-averaged molecular weights (*M_n_*), weigh-averaged molecular weights (*M_w_*), and polydispersity index (*M_w_/M_n_*) of the TPU grades. To carry out this measurement, a dilute solution of 1 mg/mL of synthesized TPU in tetrahydrofuran (THF) was prepared and stirred for 2 h. After that, the TPU solution in THF was filtered through a 0.2 μm polyamide filter. Each TPU was measured three times to obtain an average of the GPC parameters.

#### 2.5.3. Differential Scanning Calorimetry

Differential scanning calorimetry (DSC) was made with a DSC30 Mettler Toledo Instrument. All the experiments were carried out under a nitrogen atmosphere at a heating rate of 10 °C/min from −90 to 220 °C. Samples of about 8 mg were enclosed in aluminum pans. Glass transition (*T_g_*) of soft and hard segments (*T_g,SS_* and *T_g,HS_*, respectively) and melting temperature (*T_m_*) were determined by DSC. The crystallinity (*X_c_*) was estimated using Equation (1). Δ*H_m_* is the enthalpy of crystallization per gram of the sample, and Δ*H_m_*_,0_ the enthalpy of crystallization per gram of 100% crystalline TPU. The latter was taken as 155 J/g.

(1) $$X_{C}\left(\%\right)=\frac{\Delta H_{m}}{\Delta H_{m,0}}\times 100$$

#### 2.5.4. Shore Hardness

Shore D hardness tenting was determined, according to UNE-EN ISO [38]. The measurements were taken for 1 s at room temperature. Three samples of each material from the compression molded sheet with dimensions 10 × 10 × 2 mm^3^ were employed.

### 2.6. Characterization of PMMA/TPU Blends and Cellular Materials

#### 2.6.1. PMMA/TPU Blends Nanostructuration

The nanostructuration of the solid samples was analyzed using a scanning electron microscope (SEM) (FlexSEM 1000 VP-SEM, Hitachi High-Tech, Tokyo, Japan). Samples were cooled in liquid nitrogen and fractured in a transversal direction to the extrusion direction. Afterward, a chemical etching was performed using tetrahydrofuran (THF) for 30 min. THF dissolves faster the TPU phase than the PMMA, revealing the possible nanostructure of the samples. After the etching, the samples were coated with gold using a sputter coater (model SCD 005, Balzers Union, Balzers, Liechtenstein). A tool based on the software ImageJ/FIJI [39] was used to quantify the structural parameters. TPU domain density (*N_d_*) was determined according to Equation (2), where *A* is the analyzed area and *n_d_* is the number of domains in that area. In every region, more than 150 domains were analyzed. Moreover, the average domain size (*ϕ_d_*) was obtained by measuring the diameter of the domains and obtaining an average value.

(2) Nd=(ndA)32

#### 2.6.2. Density

On the one hand, density of the solid samples was measured with a gas pycnometer (model AccuPyc II 1340, Micromeritics). On the other hand, density of the cellular materials was determined with the water-displacement method based on Archimedes’ principle. A density determination kit for an AT261 Mettler-Toledo balance was used for this purpose. Some of the foamed samples present a graded cellular structure, as shown schematically in Figure S2 (Supplementary Information), with a solid skin on the surface (approximately 5 µm in thickness), a transition region where cells are microcellular and a core region in which the cells are nanocellular. To account for this heterogeneity, the density of the foamed samples was determined twice, density of the sample as produced (global density) and after removing the skin and transition regions with a polisher (model LaboPOl2-LaboForce3, Struers, Struers, Denmark) (details can be found elsewhere [30] and in the Supplementary Information Section S2) (core density). The global relative density (*ρ_r,g_*) was calculated according Equation (3) by dividing the density of the foamed sample by the density of the solid. The relative density of the homogeneous core (*ρ_r,c_*) was determined by dividing the density of the core by the density of the solid (Equation (4)).

(3) $$Global\;Relative\;Density=\frac{Global\;Density}{Solid\;PMMA\;Density}$$

(4) $$\;Core\;Relative\;Density=\frac{Core\;Density}{Solid\;PMMA\;Density}$$

#### 2.6.3. Cellular Structure

The cellular structure of the samples was analyzed using a scanning electron microscope (FlexSEM 1000 VP-SEM, Hitachi High-Tech, Tokyo, Japan). Samples were cooled in liquid nitrogen and fractured to maintain the cellular structure for the microscopic visualization. They were also coated with gold using a sputter coater (model SCD 005, Balzers Union, Balzers, Liechtenstein). Several parameters were measured in order to obtain a complete analysis of the cellular structure. Due to the gradient structure of the samples, SEM images were taken at different distances along the sample radius (in a transversal direction to the extrusion direction), and the structure was analyzed along the sample radius, and particularly in the nanocellular core. A tool based on the software ImageJ/FIJI [39] was used to quantify the structural parameters. Firstly, the average cell size in 3D (*ϕ*_3*D*_), the cell size distribution, and the standard deviation coefficient of the cell size distribution (SD) were obtained (3D values were obtained by multiplying the 2D values measured in the image by the correction factor 1.273 [39]). The parameter SD/*ϕ*_3*D*_ (normalized standard deviation coefficient) was calculated as an indicator of the homogeneity of the cellular structure. This parameter is used for comparison between materials with different cell sizes. Cell density (*N_v_*) was determined using Kumar’s theoretical approximation [40] according to Equation (5), where *A* is the analyzed area and *n* is the number of cells in that area. In every region, more than 200 cells were analyzed.

(5) Nv=(nA)32

In the core of the sample that presents homogeneous cells, the cell nucleation density (*N*_0_) was determined using Equation (6) using the relative density of the core (*ρ_r,c_*).

(6) $$N_{0}=\frac{N_{v}}{\rho_{r,c}}$$

Moreover, some of the samples presented a bimodal structure with micrometric pores among the nanocellular structure (in the core region). The fraction of area occupied by the micrometric cells in the SEM images has been measured to characterize these samples (*A_microcells_*). The criterion to classify a cell as micrometric was that a cell is micrometric when it is larger than five times the average cell size of the nanocellular region of that material.

## 3. Results and Discussion

### 3.1. Characterization of the TPU Grades

**Figure 2:**
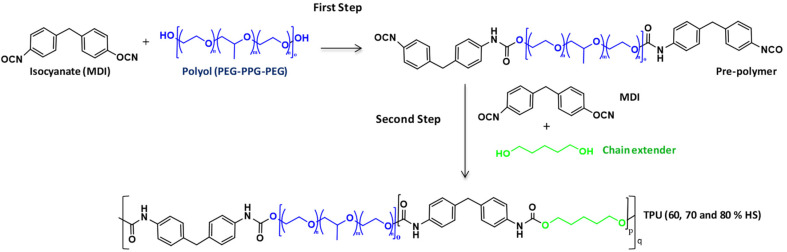
Reactions involved in the synthesis of TPU grades.

Three TPU polymers with variable HS content were used in this study (60%, 70%, and 80% of HS content). These TPUs were produced by the prepolymer process, which consists in two steps, as shown in Figure 2. In the first step, the macrodiol reacts with an excess of the diisocyanate to generate an isocyanate-terminated prepolymer. Then, the isocyanate-terminated prepolymer reacts with the chain extender in the presence of a solvent and catalyst, generating the high molecular weight TPU. Thus, these TPUs consist of linear segmented block copolymers composed of alternating soft segments (SS) from the diisocyanate and the chain extender and hard segments (HS) from the macrodiol chains. The thermodynamic incompatibility between SS and HS produces a two-phase microstructure, in which the HS segregate into semicrystalline domains through physical cross-links, whereas the SS forms amorphous domains in which the HS are dispersed [41]. Thus, HS offers rigidity, and SS provides flexibility to the TPU materials. In this study, the chemistry was varied to synthesize TPUs with high HS content.

The main characteristics of the synthesized TPU grades were characterized and collected in Table 2. The density (*ρ*) slightly increases when the HS content increases in the TPU grades. In the DSC thermograms, the glass transition temperature of soft segments (*T_g,SS_*) is only detected for the lowest content (60% of HS content) since the rest of the materials have a very low content of soft segments. However, the glass transition temperature of hard segments *(T_g,HS_*) is observed for all HS contents achieving values almost equal. Moreover, the melting temperature (*T_m_*) increases up to 6 °C when the HS content is increased from 60% to 80%. Moreover, the higher the HS content, the higher crystallinity (*X_c_*). GPC results show that the number-average molecular weight (*M_n_*) and weight-average molecular weight (*M_w_*) increase with the HS content. This increase in molecular weight is very high, from 60% to 70% of HS content, but it is very small, from 70% to 80%. The polydispersity is close to 2 in all TPU grades.

On the other hand, it is observed that the hardness gets higher with HS content. Moreover, the three TPUs grades containing a high HS content are classified in the Shore D hardness scale (referred to rigid types of TPU). The commercial TPU used in the two previous PMMA/TPU works [29,30] on nanocellular polymers had a hardness of 83 A. They are classified in the Shore A hardness scale which belongs to more flexible types of TPU (with low HS contents). Therefore, the TPUs synthesized and used in this study have a large hardness than those used in previous papers.

In the present study, TPUs with a high HS content were selected with the idea of improving dispersion in PMMA and, as a consequence, promote more intense cell nucleation. Several reasons could justify this improved dispersion. The increase of HS content supposes a decrease in macrodiol (SS) content, which is the most apolar part of the TPU. Moreover, it supposes an increase of the urethane linkages generated by the reaction of diisocyanate and chain extender diols (HS), which is the polar part of the TPU (Figure 2). Therefore, improved dispersion of the TPU in the PMMA is expected due to the higher polar structure. Moreover, increasing the HS content generates TPUs with high molecular weights (Table 2), more similar to the PMMA, which could also improve the dispersion [42]. To summarizing, the increase in HS contents is expected to promote more cell nucleation in nanocellular PMMA for the higher polarity and molecular weight of the TPU.

### 3.2. Nanostructuration of the PMMA/TPU Blends

**Figure 3:**
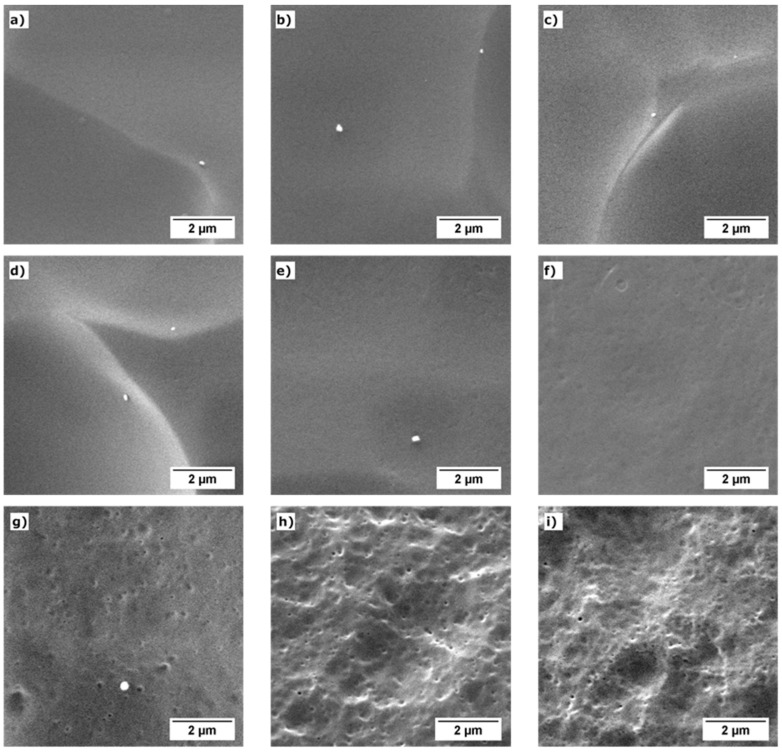
Representative SEM images of the chemical etched materials taken at the fractured surface center of the solid sample: (**a**) 0.5_60HS, (**b**) 0.5_70HS, (**c**) 0.5_80HS, (**d**) 2_60HS, (**e**) 2_70HS, (**f**) 2_80HS, (**g**) 5_60HS, (**h**) 5_70HS, and (**i**) 5_80HS.

Figure 3 shows representative SEM images of the solid materials after the chemical etching, taken at the center of the solid samples’ fracture surface (transversal direction to the extrusion direction). It is observed that at low TPU contents (0.5 wt% and 2 wt%), the etching and later gold coating do not reveal the TPU phase, except for the sample 2_80HS, where the nanostructuration can be intuited (the holes correspond to the TPU phase that is dissolved during the etching). One possible reason is that the size of the TPU domains is very small, and as the THF is also partially etching the PMMA, the holes left by the TPU phase may end up being removed. Another possible reason is that during the later gold coating process, the remaining holes are covered. Up to the precision of this method, there are no significant differences in the nanostructuration observed through the sample thickness.

At 5 wt% of TPU, the etching clearly shows the nanostructuration. Table 3 summarizes the nanostructuration characteristics of the three samples with 5 wt% of TPU (average domain size and domain density). As seen in the images, TPU forms nanometric spherical domains. As previously commented, note that we are assuming that the size of the domains is equal to the size of the holes. Then, the measured TPU domains size (Table 3) could be higher than the real size of the TPU domains.

It is observed that increasing the HS content on the TPU increases the domain density while the domain size is reduced. As previously commented, this effect may be due to the higher polarity and the higher molecular weight of the TPU as the HS content increases. Higher molecular weights are associated with higher viscosities, and according to the model proposed by Wu [42], when a polymer of viscosity *η_d_* is dispersed by extrusion in a matrix of viscosity *η_m_* (with *η_m_* > *η_d_* because the PMMA (*M_n_* = 45,000 g/mol and *M_w_* = 84,000 g/mol) presents higher molecular weight than the TPU (see Table 2)), the dispersed phase will form domains of size *d* determined by Equation (7), where *σ* is the interphase surface tension, and $\dot{\gamma }$ the extruder screws shear velocity. So, according to this equation, for a fixed polymer matrix and extrusion conditions, the size of the domains will decrease by increasing the dispersed polymer’s viscosity (molecular weight). For a fixed amount of dispersed phase, a smaller domain size implies a higher domain density. This theoretical behavior matches with the results of Table 3 and was also observed in other systems [27]. The difference in TPU domain size between blends with TPU with a 60% of HS amount and the one with 70% of HS (160 nm versus 104 nm) is greater than the difference between the TPU grades with 70% and 80% of HS (104 nm versus 95 nm). The reason is that these two TPU grades present more similar molecular weights (Table 2).

(7) $$d=\frac{\sigma \cdot 4\;\;}{\eta_{m}^{0.16}\cdot \dot{\gamma }\cdot \eta_{d}^{0.84}}$$

The order of magnitude of the obtained results for the nanostructuration of the PMMA/TPU blends is of the same order of magnitude as the values observed in the literature. Wang et al. [29] studied the nanostructuration using TEM, reporting TPU domain sizes between 42 nm and 214 nm and domain densities between 9.2 · 10^13^ and 1.5 · 10^14^ domains/cm^3^ as the TPU concentration increases from 1 wt% to 20 wt%. Meanwhile, our previous work [30] reported an average domain size of 89 nm and a domain density of 3.3 · 10^13^ domains/cm^3^ for a sample with a TPU content of 2 wt%, employing the same methodology used in this work to study the nanostructuration.

### 3.3. Effect of the Concentration of TPU and of the Fraction of HS

All the PMMA/TPU samples produced in this work (saturated at 15 MPa and 25 °C and foamed at 90 °C for 1 min) show a graded structure along the radius direction (an example of the morphology of the foamed samples and the effect of the addition of TPU is shown in Supplementary Information Section S3 (Figures S3 and S4)). The graded structure of the samples under study is characterized by decreasing cell size from the surface to the center of the sample. Depending on the sample, the core region occupies around 30%–40% of the sample radius, corresponding to 10%–15% of the total foam volume. This gradient structure was reported in our previous work [30]. Given the pressure-dependence of the nucleation in the TPU domains, we hypothesized that the graded structure formation is caused by a non-constant gas concentration profile in the samples due to the desorption of gas before the foaming step. This hypothesis was now tested and verified by performing experiments with a pre-cooling step autoclave before the pressure release to delay the diffusion process (see details of this additional experiment in Supplementary Information Section S4 (Figures S5–S8 and Table S1). In the experiments with a pre-cooling step, in which the gas desorption is slower, the cell size gradient is smoother, proving that the gradient structure is indeed due to the gas concentration profile in the samples (Supplementary information S4.1 (Figures S5 and S6 and Table S1)).

Table 4 summarizes the global and core relative densities of the foamed samples produced with the different PMMA/TPU blends and the pure PMMA. The two quantities were measured as explained in Section 2.6.2. The gradient cell size leads to a difference between the global density (*ρ_r,g_*) and the core density (*ρ_r,c_*). PMMA/TPU materials present very low global relative densities ranging from 0.11 to 0.15. Meanwhile, the core relative density is around 50% higher than the global relative density, in the range of 0.15–0.21. No trends between the relative densities and the influence of the TPU or HS content are observed. In fact, the values of all densities are within a very short range.

**Figure 4:**
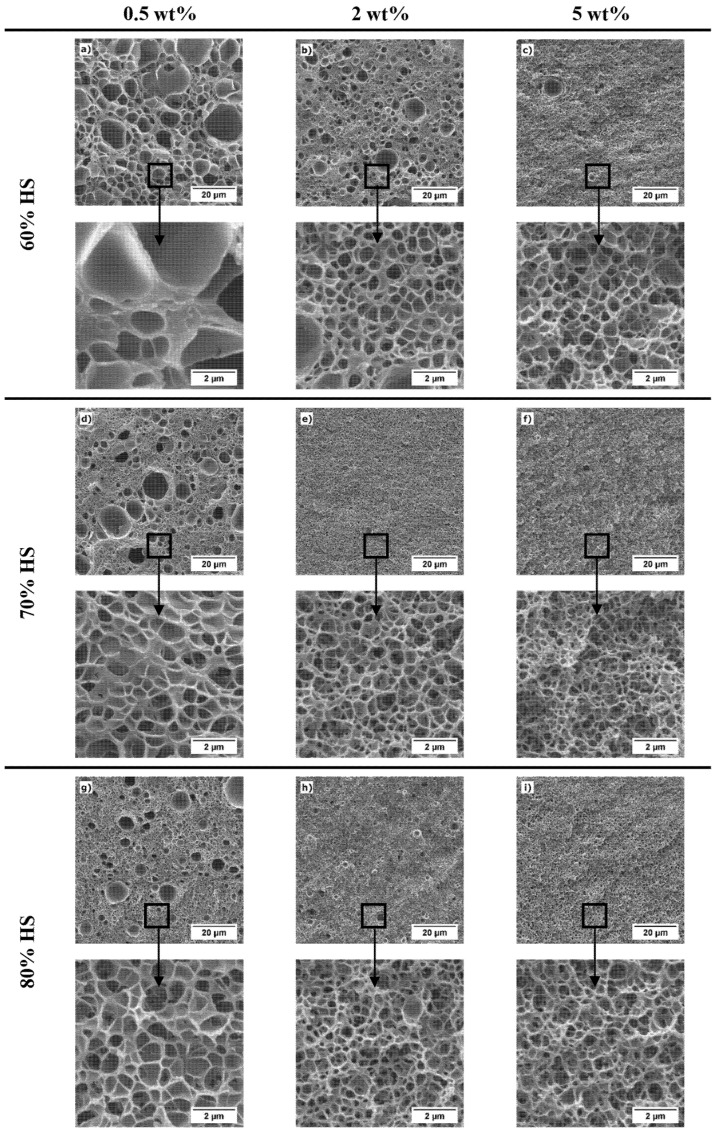
SEM images of the core of the PMMA/TPU samples saturated at 15 MPa and 25 °C and foamed at 90 °C for 1 min: (**a**) 0.5_60HS, (**b**) 2_60HS, (**c**) 5_60HS, (**d**) 0.5_70HS, (**e**) 2_70HS, (**f**) 5_70HS, (**g**) 0.5_80HS, (**h**) 2_80HS, and (**i**) 5_80HS. The second row of each series correspond to high magnification images.

From now on, the analysis presented is focused on the homogeneous core of the samples. Figure 4 shows the SEM images of the core region of the PMMA/TPU samples produced in this work (saturated at 15 MPa and 25 °C and foamed at 90 °C for 1 min). Table 5 summarizes the data obtained after the cellular structure characterization, such as cell size (*ϕ*_3*D*_) and cell nucleation density (*N*_0_) of the nanocellular region. In addition, some samples show bigger cells among the nanocellular structure (i.e., a bimodal cell size distribution). Then, to account for the bimodality, the fraction of area occupied by micrometric cells in the core region (*A_microcells_*) and the cell size of the micrometric cells (*ϕ_microcells_*) is also included in Table 5.

The material 0.5_60HS shows a microcellular wide cell size distribution in the core (mean cell size of 1417 nm and SD/*ϕ*_3*D*_ of 1.08). In contrast, the rest of the materials present a nanocellular structure with an acceptable homogeneity since the parameter SD/*ϕ*_3*D*_ takes values between 0.45 and 0.65 (Table 5). However, some of the nanocellular samples present a bimodal core structure, with microcellular cells of approximately 5 µm around the nanocells (Table 5). The area occupied by these cells is reduced as the TPU content increases and the fraction of HS of the TPU increases. For example, 0.5_70HS present 28% of the area occupied by microcellular cells versus the 13% of the sample 0.5_80HS. For the highest content of TPU (5 wt%), all the samples are monomodal and nanocellular (see Supplementary Information Section S5 (Figures S9 and S10) for details about the cell size distributions).

Several reasons might explain the bimodality. First, it may be due to a heterogeneous dispersion of the TPU phase, but this has not been observed in the solid samples (Figure 3). On the other hand, samples suffer a pre-foaming before the foaming step (i.e., they start growing before the thermal batch foaming (see Section 2.3)). Therefore, some of the cells might begin growing during the desorption step (the larger ones) and the rest (the nanometric cells) during the foaming in the thermal batch. So, not all the cells grow simultaneously in the water bath, causing a double population in the core. A test with a cooling step before the pressure release has been carried out to reduce the pre-foaming (see Supplementary Information Section S4.2 (Figures S7 and S8) for details about this experiment and its effect on the bimodality). In this experiment, we proved that the bimodality is reduced by preventing pre-foaming. Then, we concluded that pre-foaming is one of the main causes of bimodality. In the paper of Wang et al. [29] they use a cooling step before the pressure release; its purpose was to suppress the cell nucleation after depressurization (i.e., preventing pre-foaming), allowing a more homogeneous cellular structure.

**Figure 5:**
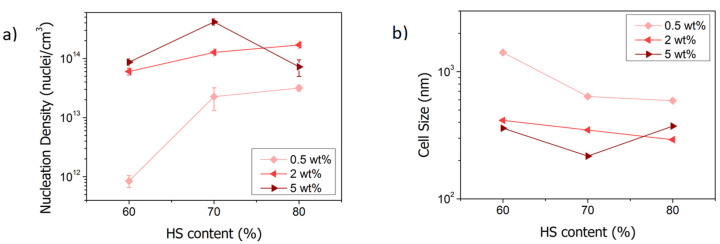
(**a**) Cell nucleation density and (**b**) cell size as a function of the HS content.

Regarding the analysis of the nanocellular region of the core, the evolution of the cell nucleation density (Figure 5a) and cell size (Figure 5b) is represented as a function of the amount of HS in the TPU. The TPU can act as a nucleating agent due to the semicrystalline domains formed by the HS promoting cell nucleation [43,44,45,46]. Considering the encouragement of cell nucleation in nanocellular PMMA, the higher content of HS in the synthesized TPUs looks to increase the effectiveness as a nucleating agent of the TPU. For instance, for the content of 0.5 wt%, increasing the amount of HS on the TPU increases de cell nucleation density and reduces the cell size. Specifically, between the contents of 60% and 70% of HS, there is a difference of more than 20 times in the cell nucleation density (8.5·10^11^ nuclei/cm^3^ versus 2.3·10^13^ nuclei/cm^3^), and the cell size is reduced to half, from 1417 nm to 638 nm (Table 5). At 80% of HS content, a cell nucleation density of 3.2·10^13^ nuclei/cm^3^ and an average cell size of 591 nm are reached. The trend is the same for a TPU content of 2 wt%, achieving a cell nucleation density of 1.7·10^14^ nuclei/cm^3^ and an average cell size of 292 nm for the TPU containing 80% of HS. By last, for the content of 5 wt%, the cell nucleation density increases (from 8.7·10^13^ nuclei/cm^3^ to 4.1·10^14^ nuclei/cm^3^), and the cell size decrease (from 359 nm to 217 nm) when the amount of HS increases from 60% to 70%. However, between the contents of 70% HS and 80% HS, the cell nucleation density is reduced, and the cell size increases, achieving 7.2·10^13^ nuclei/cm^3^ and 373 nm content (Table 5).

**Figure 6:**
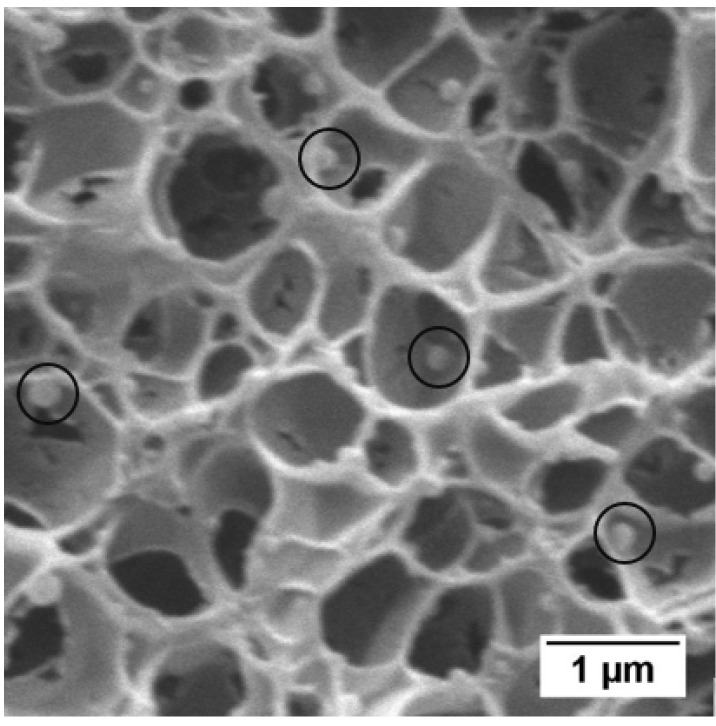
TPU nanostructuration (5_80HS).

This unexpected result may be due to different reasons. On the one hand, an excessive number of generated nuclei lead to structural degeneration mechanisms such as coalescence. On the other hand, an excess of TPU content produces agglomeration of the TPU dispersed phase during the extrusion or the saturation step. The cellular structure does not seem degenerated (Figure 6), so coalescence is discarded. Furthermore, in the cellular material 5_80HS, the TPU aggregates can be observed clearly (Figure 6), while they cannot be seen in other samples. Then, the domains are bigger than in other blends (domain size around 150 nm according to SEM images of the structure). However, in a previous section (Section 3.1), the nanometric domains found in the corresponding fractured surface center of the solid sample were smaller (around 90 nm, Table 3). So, our hypothesis for this particular system is that the TPU agglomerates during the saturation step. During the saturation, the polymer is in a rubber state, and an excessive number of domains close to each other may permit them to agglomerate. As a result, the effective nucleation in this system is reduced.

Therefore, the fraction of HS in the TPU (that determines the molecular weight and the viscosity of the polymer) plays an important role in the cellular structure of the PMMA/TPU samples. The cell sizes of the materials based on the TPU with lower HS content (lower viscosity) are larger than the rest and presenting smaller cell nucleation densities. Furthermore, as commented previously, there is a higher difference between the TPUs with 60% and 70% of HS than between the 70% and the 80% HS because the molecular weights of 70% and 80% HS are closer. Moreover, increasing TPU content increases the TPU domain density, which causes higher nucleation and smaller cell size.

Regarding nucleation efficiency, while pure PMMA shows a cell nucleation density of 2.3·10^11^ nuclei/cm^3^ and a cell size around 2200 nm, the samples with TPU present higher cell nucleation densities and smaller cell sizes due to the nanostructuration of the TPU phase as previously commented. For the TPU with 60% of HS, the cell nucleation density is 4 times higher at the lowest content (0.5 wt%) than the pure PMMA and reaches 8.7·10^13^ nuclei/cm^3^ at 5 wt% (around 400 times higher than PMMA) (Table 5). The TPU with an amount of HS of 70% presents the same trends, at 5 wt% of TPU content, the cell nucleation density is approximately 1800 times higher than the neat PMMA. Finally, the blends with TPU with 80% of HS follow the same trend for TPU contents between 0.5 wt% and 2 wt%, achieving the highest cell nucleation density and the smallest cell size at 2 wt% (1.7·10^14^ nuclei/cm^3^, which is 740 times the PMMA nucleation density). However, at 5 wt% of TPU, the nucleation density decreases to 7.2·10^13^ nuclei/cm^3^. As already mentioned, this may be due to the agglomeration of TPU domains during the saturation step.

To summarize, higher TPU contents and higher amounts of HS in the TPU lead to a higher density of TPU domains in the PMMA/TPU blend, resulting in higher cell nucleation densities and smaller cell sizes. As the number of domains increases, the distance among them is reduced, and therefore, there is less space for the cells to grow (resulting in lower cell sizes). Then, the use of TPUs with different characteristics allows controlling the cellular structure. In fact, this research demonstrates that the TPU chemistry can be modified to generate tailored organic nucleating agents for PMMA. It is also interesting to point out that the reduction of cell sizes reached by increasing the hard segment content without strongly affecting the density (see Table 4). Moreover, the bimodality (caused by the pre-foaming of the samples) is reduced when TPU domain density increases.

The optimum material produced in this work is 2_70HS because it combines very low relative density with a homogeneous nanocellular core, showing a small cell size (0.107 of global relative density, 0.156 of core relative density, and average cell size of 348 nm) (Table 4 and Table 5). The obtained results can be compared with the previous works on nanocellular polymers based on PMMA/TPU. Wang et al. [29] produced a nanocellular polymer with a minimum relative density of 0.125 and a cell size of 205 nm using a TPU content of 1 wt%. However, in this paper, the samples also presented a gradient structure, and density was measured after removing the skin, but they do not specify whether the microcellular transition region is removed or not. Meanwhile, in our previous work [30], we reported a graded cellular polymer with a minimum relative density of 0.16 and a cell size of 480 nm in the nanocellular core (global density of 0.14) at a TPU content of 2 wt%. Therefore, the most promising material produced in this work (2_70HS) presents similar relative density but improves our previous cell size results. Then, the use of self-designed TPU grades with optimized molecular structure allows both a proper control of the cellular structure and the production of low-density nanocellular polymers.

## 4. Conclusions

Low-density nanocellular polymers based on PMMA/TPU blends have been produced by means of a gas dissolution foaming process. Three grades of TPU with different amounts of HS (60%, 70%, and 80%) have been synthesized to study the influence of the TPU chemistry in the nucleating effect on PMMA. Blends with TPU contents of 0.5 wt%, 2 wt%, and 5 wt% of the three TPU grades were produced by extrusion. As a result of the dispersion of the TPU in the PMMA matrix, the solid material presents a nanostructuration formed by nanometric TPU domains dispersed in the PMMA matrix. For a fixed TPU content, as the viscosity (molecular weight) of the TPU gets closer to the PMMA viscosity, the dispersion is favored (the domain density increases and the domain size is reduced).

The morphology of the PMMA/TPU cellular materials is characterized by a gradient cell size distribution from the edge of the sample towards the core. The edge of the samples shows micrometric cells, but in the core region, the addition of TPU has a strong nucleating effect in PMMA, favoring the formation of a nanocellular structure. Thus, in the core of the PMMA/TPU samples, the cell density increases in comparison to the pure PMMA, and the cell size is reduced to the nanometric scale. The global density of the samples (including the nanocellular core and the gradient structure) ranges from 0.11 to 0.15, while the density of the nanocellular core is in the range of 0.15–0.21. Then, low-density samples are produced in this paper. Some samples present a double population of cells (micro and nanocellular) in the core that is caused partially by a pre-foaming.

Regarding the influence of the TPU molecular structure, as the amount of HS in the TPU increases, the cell nucleation density increases, and the cell size is reduced. This is due to the higher molecular weight that favors the dispersion of TPU in PMMA. On the other hand, results show that by increasing the concentration of TPU, the cell nucleation density increases, and the cell size is reduced due to the increment of the number of domains of TPU dispersed in the solid. Moreover, the bimodality is reduced when TPU domain density increases because distance among them is reduced. Then, the amount of HS in the TPU and the concentration of TPU can be used as tunable parameters to control the cellular structure in the PMMA/TPU nanocellular polymers. Among the results obtained, the promising material is obtained by using a 2 wt% of the TPU with 70% of HS. This material has a homogeneous nanocellular core with a relative density of 0.156 and a cell size of 348 nm.

## Figures and Tables

**Table 1:** The amount of each component used for the first and second steps of TPU synthesis.

TPU GRADES	FIRST STEP		SECOND STEP		
	MDI Isocyanate	PEG-PPG-PEG Macrodiol	PREPOLYMER	MDI Isocyanate	1,5-PDO Chain Extender
60% HS	64.2 g, 0.257 mol	85.8 g, 0.043 mol	69.9 g	13.4 g, 0.054 mol	16.7 g, 0.160 mol
70% HS	64.2 g, 0.257 mol	85.8 g, 0.043 mol	52.5 g	28.3 g, 0.113 mol	19.2 g, 0.184 mol
80% HS	64.2 g, 0.257 mol	85.8 g, 0.043 mol	35.0 g	42.6 g, 0.170 mol	22.4 g, 0.215 mol

**Table 2:** Characteristics of the TPU polymers used in this work.

Description	*ρ* (g/cm^3^)	*T_g,SS_* (°C)	*T_g,HS_* (°C)	*T_m_* (°C)	X_c_ (%)	Hardness (Shore D)	*M_n_* (g/mol)	*M_w_* (g/mol)	Polydispersity Index
60% HS	1.192	−43.3	55.5	177.5	10.6	45.6 ± 0.5	8552 ± 1602	18,325 ± 535	2.18 ± 0.32
70% HS	1.204	-	55.5	183.5	12.8	63.2 ± 0.4	14,043 ± 1162	31,449 ± 128	2.25 ± 0.20
80% HS	1.217	-	51.3	183.4	13.4	72.4 ± 0.5	15,086 ± 2126	32,603 ± 4825	2.16 ± 0.02

**Table 3:** Nanostructuration characteristics of the three PMMA/TPU solid samples with 5 wt% of TPU produced in this work.

Material	*ϕ_d_* (nm)	SD/*ϕ_d_*	*N_d_* (Domains/cm^3^)
5_60HS	160	0.33	2.3 · 10^13^
5_70HS	104	0.19	4.0 · 10^13^
5_80HS	95	0.15	1.2 · 10^14^

**Table 4:** Global and core relative density (global and core) of the produced samples.

Sample ID	*ρ_r,g_*	*ρ_r,c_*
PMMA	0.147 ± 0.001	0.175 ± 0.002
0.5_60HS	0.143 ± 0.001	0.206 ± 0.002
2_60HS	0.138 ± 0.001	0.200 ± 0.001
5_60HS	0.148 ± 0.001	0.174 ± 0.002
0.5_70HS	0.123 ± 0.001	0.177 ± 0.001
2_70HS	0.107 ± 0.001	0.156 ± 0.005
5_70HS	0.139 ± 0.001	0.171 ± 0.002
0.5_80HS	0.110 ± 0.001	0.153 ± 0.001
2_80HS	0.134 ± 0.001	0.191 ± 0.001
5_80HS	0.152 ± 0.001	0.202 ± 0.001

**Table 5:** Cellular structure characteristics of the core of the foamed samples.

Sample ID	*N*_0_ (Nulcei/cm^3^)	*ϕ*_3*D*_ (nm)	SD/*ϕ*_3*D*_	Bimodality		
				*A_microcells_* (%)	*ϕ_microcells_* (µm)	SD/*ϕ_microcells_*
0.5_60HS	(8.5 ± 1.9) · 10^11^	1417	1.08	-	-	-
2_60HS	(6.0 ± 0.8) · 10^13^	414	0.47	33	4.4	0.75
5_60HS	(8.7 ± 0.5) · 10^13^	359	0.64	-	-	-
0.5_70HS	(2.3 ± 0.9) · 10^13^	638	0.52	28	5.8	0.63
2_70HS	(1.3 ± 0.0) · 10^14^	348	0.52	-	-	-
5_70HS	(4.1 ± 0.1) · 10^14^	217	0.57	-	-	-
0.5_80HS	(3.2 ± 0.3) · 10^13^	591	0.48	13	5.8	0.50
2_80HS	(1.7 ± 0.0) · 10^14^	292	0.49	3	3.5	0.37
5_80HS	(7.2 ± 2.2) · 10^13^	373	0.62	-	-	-

## Data Availability

Not applicable.

## Supplementary Materials

The following are available online at https://www.mdpi.com/article/10.3390/polym13183055/s1, Figure S1: Photograph of the solid samples produced in this work. For the same type of TPU the contents of TPU were 0.5 wt%, 2 wt%, and 5 wt%; Figure S2: (a) Schematic structure of the cylindrical samples obtained in this work. (b) Comparison between the as produced foamed sample and after removing the skin and transition regions; Figure S3: Representative SEM images of the cellular materials: (a) 2_70HS and (b) pure PMMA sample. Each image is taken at an increasing distance from the edge to the center of the sample along the sample radius (a.1 and b.1 NEAR THE EDGE (rd ~ 1); a.2 and b.2: rd ~ 0.75; a.3 and b.3: rd ~ 0.50; a.4 and b.4: rd ~ 0.25). Images a.4 and b.4 have been taken in the core of the samples; Figure S4: (a) Cell density and (b) cell size of the cellular samples as a function of the radial distance from the center (rd ~ 0%) to the edge (rd ~ 100%) of the 2_70HS and PMMA; Figure S5: Representative SEM images of the material 2_70HS: (a) Normal conditions and (b) Cooling step before depressurization. Each image is taken at an increasing distance from the edge to the center of the sample along the sample radius (a.1 and b.1 NEAR THE EDGE (rd ~ 1); a.2 and b.2: rd ~ 0.75; a.3 and b.3: rd ~ 0.50; a.4 and b.4: rd ~ 0.25). Images a.4, b.3, and b.4 have been taken in the core of the samples; Figure S6: (a) Cell density and (b) cell size of the as a function of the radial distance from the center (rd ~ 0%) to the edge of the sample (rd ~ 100%) of the material 2_70HS produced at normal and cooled conditions; Figure S7: SEM images of the material: (a) 0.5_60HS, (b) 2_60HS, (c) 5_60HS, (d) 0.5_70HS, (e) 2_70HS, (f) 5_70HS, (g) 0.5_80HS, (h) 2_80HS and (i) 5_80HS. Normal conditions (Left) and cooling step before depressurization (Right); Figure S8: Global and core relative density of the foamed samples: (a) normal conditions and (b) cooling step before depressurization; Figure S9: Core cell size distribution of the nanocellular region of the PMMA/TPU samples produced at normal conditions: (a) 0.5_60HS, (b) 2_60HS, (c) 5_60HS, (d) 0.5_70HS, (e) 2_70HS, (f) 5_70HS, (g) 0.5_80HS, (h) 2_80HS and (i) 5_80HS; Figure S10. Core cell size distribution of the microcellular region of the PMMA/TPU samples produced at normal conditions: (a) 2_60HS, (b) 0.5_70HS, (c) 0.5_80HS, (d) 2_80HS; Table S1: Cellular characteristics of core region of the sample 2_70HS.

## References

[1] Martín-de León J., Bernardo V., Rodríguez-Pérez M. (2019). Nanocellular Polymers: The Challenge of Creating Cells in the Nanoscale. Materials.

[2] Li Z.-Y., Zhu C.-Y., Key X.-P.Z. (2017). A theoretical and numerical study on the gas-contributed thermal conductivity in aerogel. Int. J. Heat Mass Transf..

[3] Notario B., Pinto J., Rodriguez-Perez M.A. (2016). Nanoporous polymeric materials: A new class of materials with enhanced properties. Prog. Mater. Sci..

[4] Notario B., Pinto J., Solorzano E., de Saja J.A., Dumon M., Rodríguez-Pérez M.A. (2015). Experimental validation of the Knudsen effect in nanocellular polymeric foams. Polymer.

[5] Pinto J., Notario B., Verdejo R., Dumon M., Costeux S., Rodriguez-Perez M.A. (2017). Molecular confinement of solid and gaseous phases of self-standing bulk nanoporous polymers inducing enhanced and unexpected physical properties. Polymer.

[6] Notario B., Pinto J., Rodríguez-Pérez M.A. (2015). Towards a new generation of polymeric foams: PMMA nanocellular foams with enhanced physical properties. Polymer.

[7] Xiang B., Jia Y., Lei Y., Zhang F., He J., Liu T., Luo S. (2019). Mechanical properties of microcellular and nanocellular silicone rubber foams obtained by supercritical carbon dioxide. Polym. J..

[8] Martín-de León J., Bernardo V., Rodríguez-Pérez M.Á. (2017). Key Production Parameters to Obtain Transparent Nanocellular PMMA. Macromol. Mater. Eng..

[9] Martín-de León J., Pura J.L., Bernardo V., Rodríguez-Pérez M.Á. (2019). Transparent nanocellular PMMA: Characterization and modeling of the optical properties. Polymer.

[10] Pinto J., Dumon M., Rodriguez-Perez M.A., Garcia R., Dietz C. (2014). Block Copolymers Self-Assembly Allows Obtaining Tunable Micro or Nanoporous Membranes or Depth Filters Based on PMMA; Fabrication Method and Nanostructures. J. Phys. Chem. C.

[11] Lu G.Q., Zhao X.S. (2004). Nanoporous Materials—An Overview. Nanoporous Materials: Science and Engineering.

[12] Wang G., Zhao G., Zhang L., Mu Y., Park C.B. (2018). Lightweight and tough nanocellular PP/PTFE nanocomposite foams with defect-free surfaces obtained using in situ nanofibrillation and nanocellular injection molding. Chem. Eng. J..

[13] Costeux S. (2014). CO_2_-blown nanocellular foams. J. Appl. Polym. Sci..

[14] Martín-de León J., Bernardo V., Rodríguez-Pérez M. (2016). Low Density Nanocellular Polymers Based on PMMA Produced by Gas Dissolution Foaming: Fabrication and Cellular Structure Characterization. Polymers.

[15] Guo H., Nicolae A., Kumar V. (2015). Solid-state poly(methyl methacrylate) (PMMA) nanofoams. Part II: Low-temperature solid-state process space using CO2 and the resulting morphologies. Polymer.

[16] Yoon T.J., Kong W., Kwon D.E., Park B.K., Lee W.I., Lee Y.W. (2017). Preparation of solid-state micro- and nanocellular acrylonitrile-butadiene-styrene (ABS) foams using sub- and supercritical CO2 as blowing agents. J. Supercrit. Fluids.

[17] Costeux S., Zhu L. (2013). Low density thermoplastic nanofoams nucleated by nanoparticles. Polymer.

[18] Bernardo V., Martín-de León J., Laguna-Gutiérrez E., Rodríguez-Pérez M.Á. (2017). PMMA-sepiolite nanocomposites as new promising materials for the production of nanocellular polymers. Eur. Polym. J..

[19] Xiao S.P., Huang H.X. (2019). Generation of nanocellular TPU/reduced graphene oxide nanocomposite foams with high cell density by manipulating viscoelasticity. Polymer.

[20] Jahani D., Azimi H., Nazari A. (2019). An experimental study on the micro- And nanocellular foaming of polystyrene/poly(methyl methacrylate) blend composites. J. Polym. Eng..

[21] Forest C., Chaumont P., Cassagnau P., Swoboda B., Sonntag P. (2015). CO_2_ Nano-foaming of nanostructured PMMA. Polymer.

[22] Nofar M., Büşra Küçük E., Batı B. (2019). Effect of hard segment content on the microcellular foaming behavior of TPU using supercritical CO2. J. Supercrit. Fluids.

[23] Liu S., Yin S., Duvigneau J., Vancso G.J. (2020). Bubble Seeding Nanocavities: Multiple Polymer Foam Cell Nucleation by Polydimethylsiloxane-Grafted Designer Silica Nanoparticles. ACS Nano.

[24] Liu Q., Zhu Y., Yang G., Yang Q. (2008). Nucleation thermodynamics inside micro/nanocavity. J. Mater. Sci. Technol..

[25] Yeh S.-K., Chen Y.-R., Kang T.-W., Tseng T.-J., Peng S.-P., Chu C.-C., Rwei S.-P., Guo W.-J. (2018). Different approaches for creating nanocellular TPU foams by supercritical CO2 foaming. J. Polym. Res..

[26] Ni J., Yu K., Zhou H., Mi J., Chen S., Wang X. (2020). Morphological evolution of PLA foam from microcellular to nanocellular induced by cold crystallization assisted by supercritical CO2. J. Supercrit. Fluids.

[27] Bernardo V., Martin-de Leon J., Laguna-Gutierrez E., Catelani T., Pinto J., Athanassiou A., Rodriguez-Perez M.A. (2018). Understanding the role of MAM molecular weight in the production of PMMA/MAM nanocellular polymers. Polymer.

[28] Bernardo V., Martin-de Leon J., Pinto J., Catelani T., Athanassiou A., Rodriguez-Perez M.A. (2019). Low-density PMMA/MAM nanocellular polymers using low MAM contents: Production and characterization. Polymer.

[29] Wang G., Zhao J., Mark L.H., Wang G., Yu K., Wang C., Park C.B., Zhao G. (2017). Ultra-tough and super thermal-insulation nanocellular PMMA/TPU. Chem. Eng. J..

[30] Bernardo V., Martin-de Leon J., Sanchez-Calderon I., Laguna-Gutierrez E., Rodriguez-Perez M.A. (2019). Nanocellular Polymers with a Gradient Cellular Structure Based on Poly(methyl methacrylate)/Thermoplastic Polyurethane Blends Produced by Gas Dissolution Foaming. Macromol. Mater. Eng..

[31] Ghaffari Mosanenzadeh S., Naguib H.E., Park C.B., Atalla N. (2015). Design and development of novel bio-based functionally graded foams for enhanced acoustic capabilities. J. Mater. Sci..

[32] Monnereau L., Urbanczyk L., Thomassin J.-M., Pardoen T., Bailly C., Huynen I., Jérôme C., Detrembleur C. (2015). Gradient foaming of polycarbonate/carbon nanotube based nanocomposites with supercritical carbon dioxide and their EMI shielding performances. Polymer.

[33] Pinto J., Morselli D., Bernardo V., Notario B., Fragouli D., Rodriguez-Perez M.A., Athanassiou A. (2017). Nanoporous PMMA foams with templated pore size obtained by localized in situ synthesis of nanoparticles and CO 2 foaming. Polymer.

[34] Trofa M., Di Maio E., Maffettone P.L. (2019). Multi-graded foams upon time-dependent exposition to blowing agent. Chem. Eng. J..

[35] Kumar V., Suh N.P. (1990). A process for making microcellular thermoplastic parts. Polym. Eng. Sci..

[36] Pinto J., Dumon M., Pedros M., Reglero J., Rodriguez-Perez M.A. (2014). Nanocellular CO2 foaming of PMMA assisted by block copolymer nanostructuration. Chem. Eng. J..

[37] Tammaro D., Iannace S., Di Maio E. (2017). Insight into bubble nucleation at high-pressure drop rate. J. Cell. Plast..

[38] UNE-EN ISO 868:2003 (2003). Determinación de la Dureza de Indentación por Medio de un Durómetro (Dureza Shore).

[39] Pinto J., Solórzano E., Rodriguez-Perez M.A., de Saja J.A. (2013). Characterization of the cellular structure based on user-interactive image analysis procedures. J. Cell. Plast..

[40] Kumar V. (1988). Process synthesis for manufacturing microcellular thermoplastic parts. Ph.D. Thesis.

[41] Prisacariu C. (2011). Polyurethane Elastomers.

[42] Wu S. (1987). Formation of dispersed phase in incompatible polymer blends: Interfacial and rheological effects. Polym. Eng. Sci..

[43] Wong A., Guo Y., Parka C.B. (2013). Fundamental mechanisms of cell nucleation in polypropylene foaming with supercritical carbon dioxide—Effects of extensional stresses and crystals. J. Supercrit. Fluids.

[44] Lips P.A.M., Velthoen I.W., Dijkstra P.J., Wessling M., Feijen J. (2005). Gas foaming of segmented poly(ester amide) films. Polymer.

[45] Mihai M., Huneault M.A., Favis B.D. (2010). Rheology and extrusion foaming of chain-branched poly(lactic acid). Polym. Eng. Sci..

[46] Taki K., Kitano D., Ohshima M. (2011). Effect of growing crystalline phase on bubble nucleation in poly(L -lactide)/CO2 batch foaming. Ind. Eng. Chem. Res..

