# Supplementary material for: Effect of the Molecular Structure of TPU on the Cellular Structure of Nanocellular Polymers Based on PMMA/TPU Blends
Source: Polymers (Basel). 2021 Sep 10;13(18):3055. doi: 10.3390/polym13183055 (PMC8473334; doi:10.3390/polym13183055)
Supplement: Supplementary file 1 [file polymers-13-03055-s001.zip › polymers-1320072-supplementary.pdf]

polymers
MDPI

# Supplementary Information

# Effect of the molecular structure of TPU on the cellular structure of nanocellular polymers based on PMMA/TPU blends

**Ismael Sánchez-Calderón [1], Victoria Bernardo [2], Mercedes Santiago-Calvo [1], Haneen Naji [3], Alberto Saiani [3] and Miguel Ángel Rodríguez-Pérez [1,4].**

1 Cellular Materials Laboratory (CellMat), Condensed Matter Physics Department, University of Valladolid, Campus Miguel Delibes, Paseo de Belén nº7, 47011, Valladolid, Spain
2 CellMat Technologies S.L. Paseo de Belén 9-A 47011, Valladolid, Spain
3 School of Materials, The University of Manchester, Oxford Road, Manchester M13 9PL, UK
4 BioEcoUVA Research Institute on Bioeconomy, University of Valladolid, Spain

## S1. PMMA/TPU blends

Figure S1 shows a photograph of examples of the solid samples produced in this work. As seen in Figure S1, by increasing the TPU content, the samples become opaquer. Also, the samples become opaquer for a fixed TPU content by increasing the fraction of HS in the TPU. These effects are due to the dispersion of the TPU in the PMMA matrix. As shown in the main text, increasing the TPU content and the fraction of HS in the TPU leads to a higher TPU domains density, resulting in higher light diffraction, reducing the transparency.

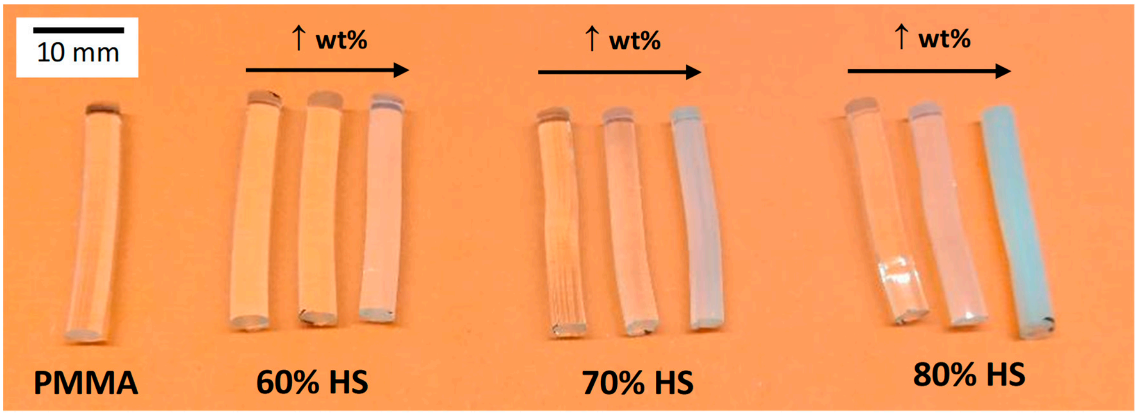


**Figure S1.** Photograph of the solid samples produced in this work. For the same type of TPU the contents of TPU were 0.5 wt%, 2 wt%, and 5 wt%.

## S2. Graded cellular structure

Some of the samples of this work present a graded cellular structure, as shown schematically in Figure S2a. The graded cellular structure is characterized by a solid skin on the surface of the samples (approximately 5 μm in thickens), a transition region where cells are microcellular, and a core region in which the cells are nanocellular. The thickness of the transition region, named as "gradient distance" (Equation (1)), is the distance between the edge of the sample ($r_{sample}$) and the homogeneous nanocellular core ($r_{core}$). Then, to account for this heterogeneity, the density of the foamed samples was determined twice. Firstly, the density of the complete foamed sample (as removed from the thermal bath and after waiting enough time to assure that the remaining gas had diffused out) was determined, and using this density, the global relative density was calculated ($\rho_{r,g}$) by using Equation (2). Secondly, the skin and transition regions (grey and red regions in Figure S2a) were removed with a polisher (model LaboPOl2-LaboForce3, Struers), the density was measured again, and with this new value, the relative density of the homogeneous core (blue region in Figure S2a) was determined ($\rho_{r,c}$) by using Equation (2). Figure S2b shows a comparison between the as-produced foamed sample (used to determine the global relative density) and after removing the skin and transition regions (used to determine the core relative density). The thickness of the transition region to be removed was determined by analyzing the cellular structure using SEM. Also, for the PMMA, which does not present a clear gradient cellular structure, we removed sample

thickness until reaching a radius of 0.75 mm. This distance is into the core of all PMMA/TPU samples being a good reference for measuring the core density.

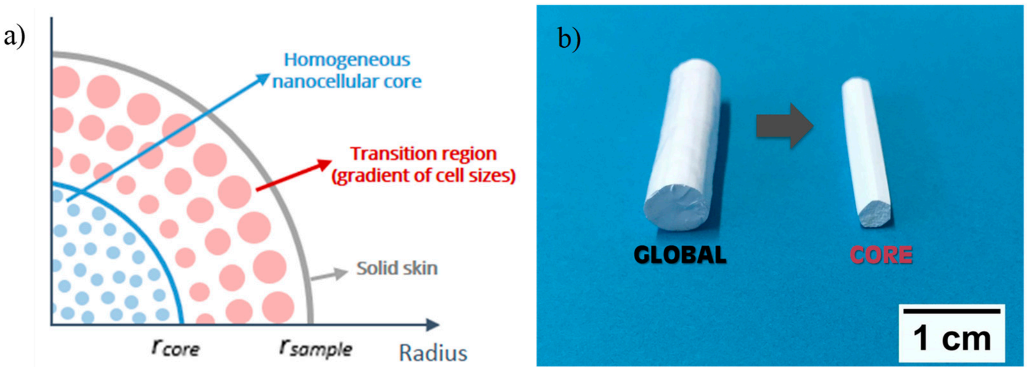


**Figure S2.** (**a**) Schematic structure of the cylindrical samples obtained in this work. (**b**) Comparison between the as produced foamed sample and after removing the skin and transition regions.

$$Gradient\ Distance = r_{sample} - r_{core} \quad (1)$$

$$Relative\ Density = \frac{Foam\ Density}{Solid\ PMMA\ Density} \quad (2)$$

## S3. Morphology of the foamed samples and effect of the addition of TPU

Figure S3 shows representative SEM images taken at increasing distances from the edge to the center of the samples of the cellular materials based on the blend 2_70HS (Figure S3a) and of the pure PMMA sample (Figure S3b). As shown in Figure S4, where the evolution of the cell density (Figure S4a) and of the cell size (Figure S4b) throughout the sample radius ($r_d$) is presented, the cellular material 2_70HS presents a gradient cellular structure. The material is microcellular near the edge and becomes nanocellular in the core. Meanwhile, the cellular structure of the pure PMMA sample is almost constant along the sample radius and is clearly heterogeneous and microcellular. Moreover, comparing Figure S3a.4 and Figure S3b.4, which correspond to the core region of each sample, it is observed that the addition of TPU has a nucleating effect in PMMA. Thus, in the sample which contains TPU, the cell density increases about $10^{13}$ cells/cm$^3$ (Figure S4a) at the time the cell size is reduced to the nanometric scale (around 350 nm) when the core of the sample is reached (Figure S4b).

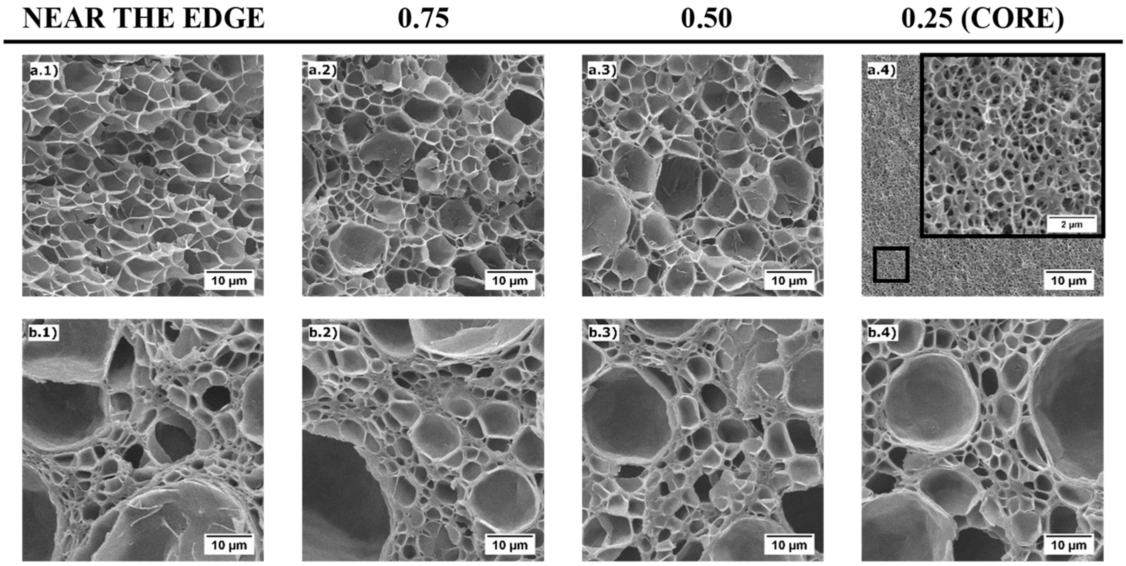


**Figure S3.** Representative SEM images of the cellular materials: (**a**) 2_70HS and (**b**) pure PMMA sample. Each image is taken at an increasing distance from the edge to the center of the sample along the sample radius ((**a.1**) and (**b.1**) NEAR THE EDGE ($r_d$~1); (**a.2**) and (**b.2**): $r_d$~0.75; (**a.3**) and (**b.3**): $r_d$~0.50; (**a.4**) and (**b.4**): $r_d$~0.25). Images a.4 and b.4 have been taken in the core of the samples.

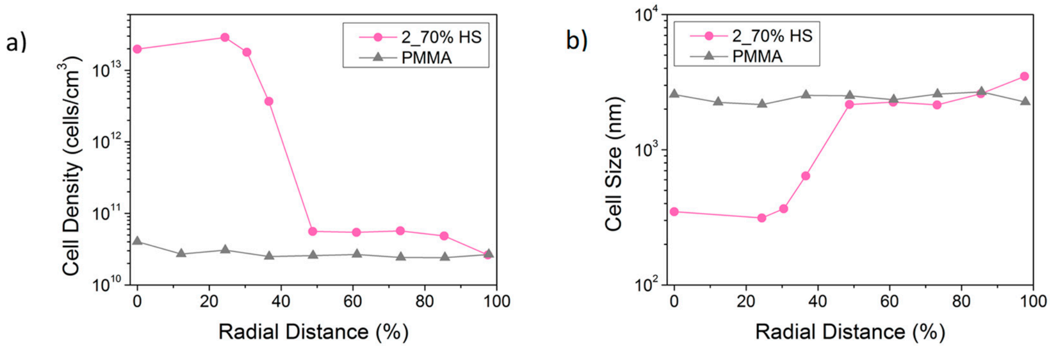


**Figure S4.** (**a**) Cell density and (**b**) cell size of the cellular samples as a function of the radial distance from the center ($r_d$~0%) to the edge ($r_d$~100%) of the 2_70HS and PMMA.

## S4. Effect of cooling the autoclave before the pressure release

An additional foaming test in which a cooling step is introduced before releasing the pressure has been carried out to study the gradient cellular structure and the bimodality observed in the PMMA/TPU foams. The cooling may allow the samples to absorb more gas near the surface since solubility increases as temperature decrease in PMMA [1]. Also, by freezing the samples before the pressure release, it is harder for the gas to diffuse outwards during depressurization because the diffusion rate will be slower at a lower temperature.

In this experiment, a cooling step before the depressurization was added to the usual gas dissolution foaming. Ice and liquid nitrogen were used to cool the autoclave for 30 min, reaching a temperature of 12 °C inside the autoclave. When the autoclave was opened, dry ice was formed (temperature of −66 °C according to the inner thermocouple). The samples included in this test were the pure PMMA sample and the PMMA/TPU blends.

### *S4.1. Analysis of the gradient cellular structure of the sample 2_70%HS*

Figure S5 shows representative SEM images taken at increasing distances from the edge to the center of the sample along the sample radius for the samples produced at normal conditions (that is, with depressurization at room temperature, Figure S5a) and with the extra cooling step (Figure S5b). Sample 2_70HS was used a reference material to show the general behavior. The quantitative analysis of the cellular structure (cell size and cell density) throughout the sample radius ($r_d$) is plotted in Figure S6. On the one hand, as already seen in the main text of this paper, the sample produced

at normal conditions presents a gradient structure from the edge to the center (Figure S5a): the cell density increases (Figure S6a), and the cell size decreases (Figure S6b). On the other hand, the cooled sample presents a gradient, but it is smoother, as shown in Figure S6. Also, a circular crown with smaller cell sizes can be observed into the gradient and near the surface (Figure S6b). Note that cooling may allow the sample to achieve more gas near the surface. Therefore, the cooling step reduces the loos of gas in the surfaces and promotes the TPU nucleation. Differences between both tests prove that the gas concentration causes the gradient structure.

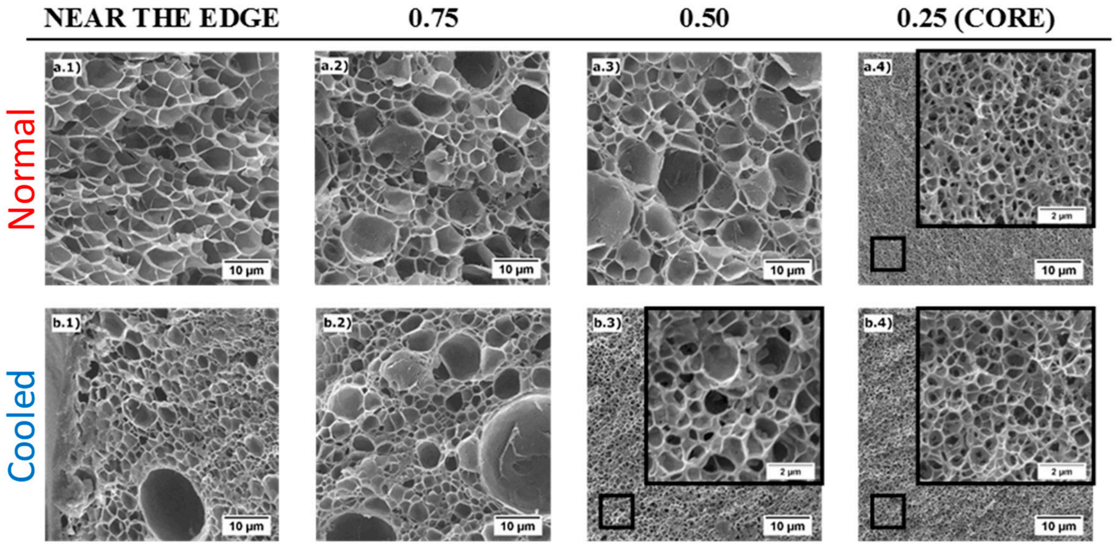


**Figure S5.** Representative SEM images of the material 2_70HS: (**a**) Normal conditions and (**b**) Cooling step before depressurization. Each image is taken at an increasing distance from the edge to the center of the sample along the sample radius ((**a.1**) and (**b.1**) NEAR THE EDGE ($r_d$~1); (**a.2**) and (**b.2**): $r_d$~0.75; (**a.3**) and (**b.3**): $r_d$~0.50; (**a.4**) and (**b.4**): $r_d$~0.25). Images (**a.4**), (**b.3**), and (**b.4**) have been taken in the core of the samples.

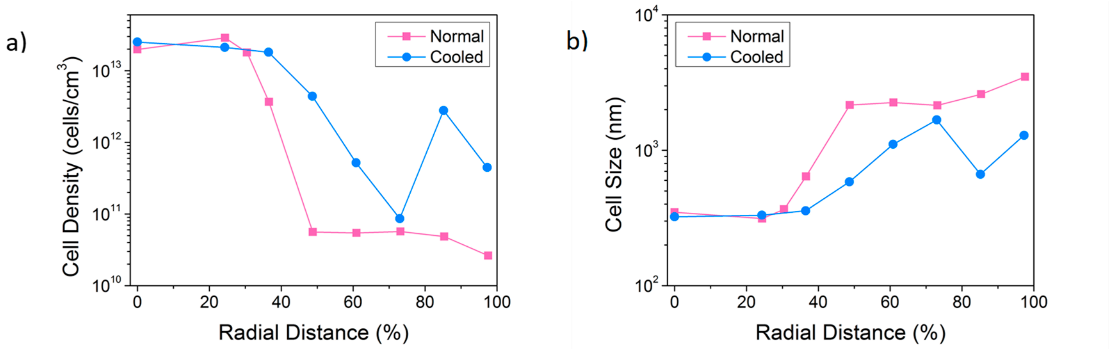


**Figure S6.** (**a**) Cell density and (**b**) cell size of the as a function of the radial distance from the center ($r_d$~0%) to the edge of the sample ($r_d$~100%) of the material 2_70HS produced at normal and cooled conditions.

Also, Table S1 summarizes the analysis of the core nanocellular region of the 2_70HS material. Firstly, the cooled sample presents higher relative densities than in normal conditions. The cooling may cause this: since the sample is cooler, it might require higher foaming times to achieve the same expansion (i.e., the optimum foaming conditions could be different for the experiments in which the cooling step was introduced). Secondly, in the cooled sample, the global relative density ($\rho_{r,g}$) and the core relative density ($\rho_{r,c}$) are almost the same (0.170 versus 0.167, respectively), indicating a smooth cell size gradient as already observed in the SEM images. The cell size is slightly smaller in the cooled test (348 nm versus 322 nm in the cooling experiment), but the cell density and the cell nucleation density in both cases are very similar. This is a reasonable result because the nucleation depends on the gas concentration (pressure), and thus, at equal pressure in the core, the same nucleation density should be reached.

**Table S1.** Cellular characteristics of core region of the sample 2_70HS.

| 2_70% HS | $\rho_{r,g}$ | $\rho_{r,c}$ | $\phi$ (nm) | SD/ $\phi$ | $N_v$ (cells/cm$^3$) | $N_0$ (nuclei/cm$^3$) |
|---|---|---|---|---|---|---|
| Normal | 0.107 | 0.156 | 348 | 0.52 | $2.0 \times 10^{13}$ | $1.3 \times 10^{14}$ |
| Cooled | 0.17 | 0.167 | 322 | 0.49 | 2.5 × 1013 | 1.5 × 1014 |

*S4.2. Analysis of the bimodality of several PMMA/TPU samples*

Besides the reduction of the thickness of the gradient structure, cooling before releasing the pressure has another effect on the structure of the PMMA/TPU samples. It is observed that the cooling step reduces the bimodality of the nanocellular core, as shown representatively in Figure S7. If poor TPU dispersion was the cause of the bimodality, both cellular structures had to be similar. However, by cooling before releasing the pressure, the bimodality is reduced. The cooling reduces the pre-foaming, improving the homogeneity of the cellular structure. The fact that bimodality still exists at the lowest contents might be due to the TPU dispersion; the content might be too low to fill the sample homogeneously.

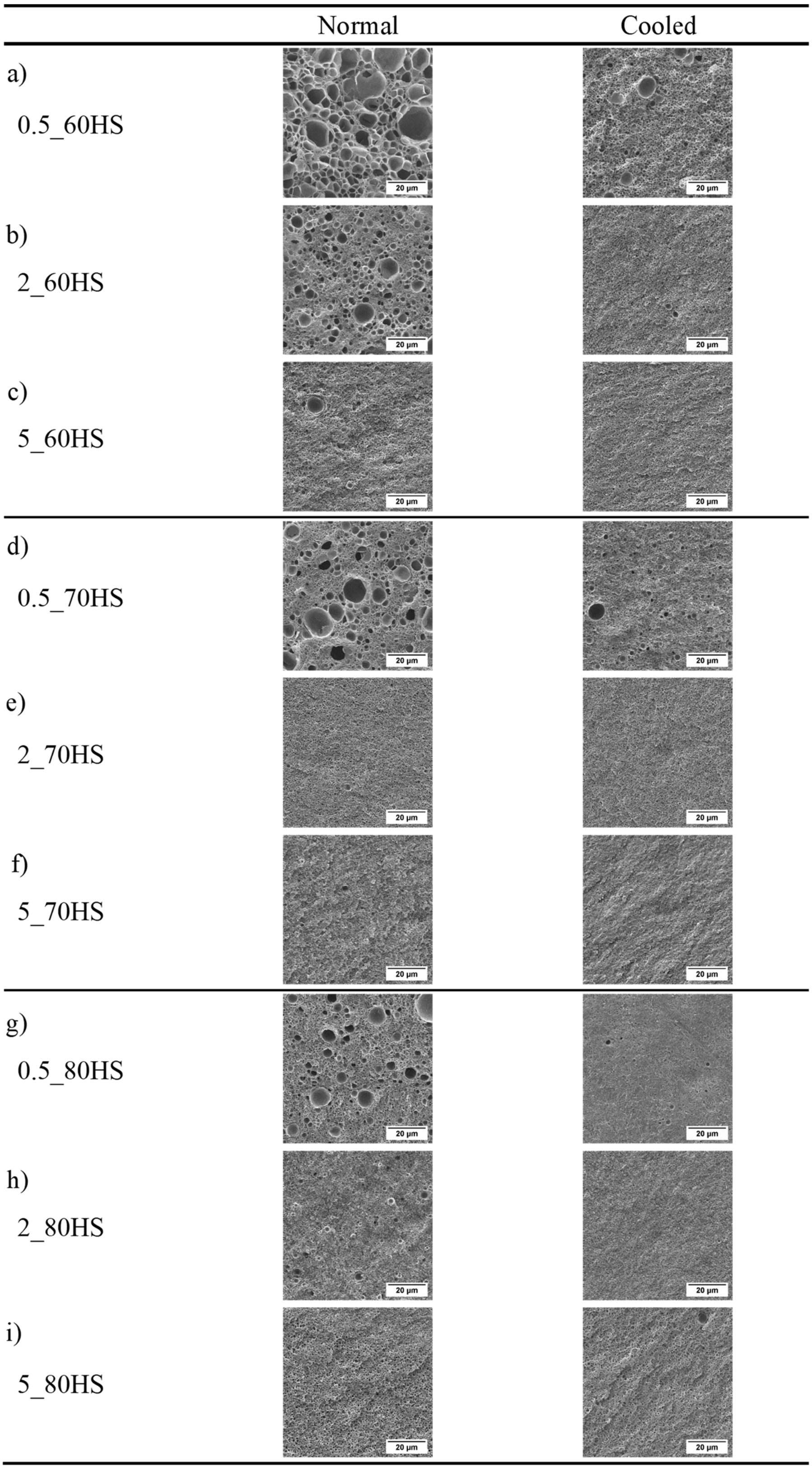


**Figure S7.** SEM images of the material: (**a**) 0.5_60HS, (**b**) 2_60HS, (**c**) 5_60HS, (**d**) 0.5_70HS, (**e**) 2_70HS, (**f**) 5_70HS, (**g**) 0.5_80HS, (**h**) 2_80HS and (**i**) 5_80HS. Normal conditions (Left) and cooling step before depressurization (Right).

Figure S8 shows the global and core relative density of the foamed samples in the normal experiment (Figure S8a) and in the cooled test (Figure S8b). It is observed that the cooled samples present higher relative densities than in normal conditions because the structure requires higher foaming times or temperatures to achieve the same expansion. As TPU

content increases, the core relative density approaches to the global relative density, even reaching lowers values for some of the samples. One possible reason is that the appearance of cells of smaller sizes near the surface increased the global relative density. While increasing the TPU content, the size of these cells decreases.

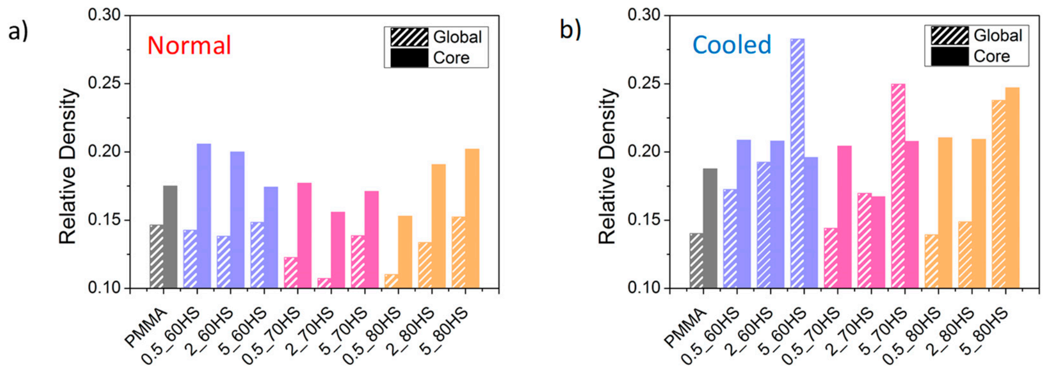


**Figure S8.** Global and core relative density of the foamed samples: (**a**) normal conditions and (**b**) cooling step before depressurization.

## S5. Cell size distribution of the PMMA/TPU samples

Figure S9 and Figure S10 show the cell size distributions of the materials produced at 15 MPa of saturation pressure (normal conditions) and foamed at 90 ºC for 1 minute. The nanocellular region is in Figure S9, while the microcellular region is in Figure S10. This information completes Table 4 (in the main text) and supports the discussion about the effect of the TPU content and the amount of HS in the TPU in the cell size.

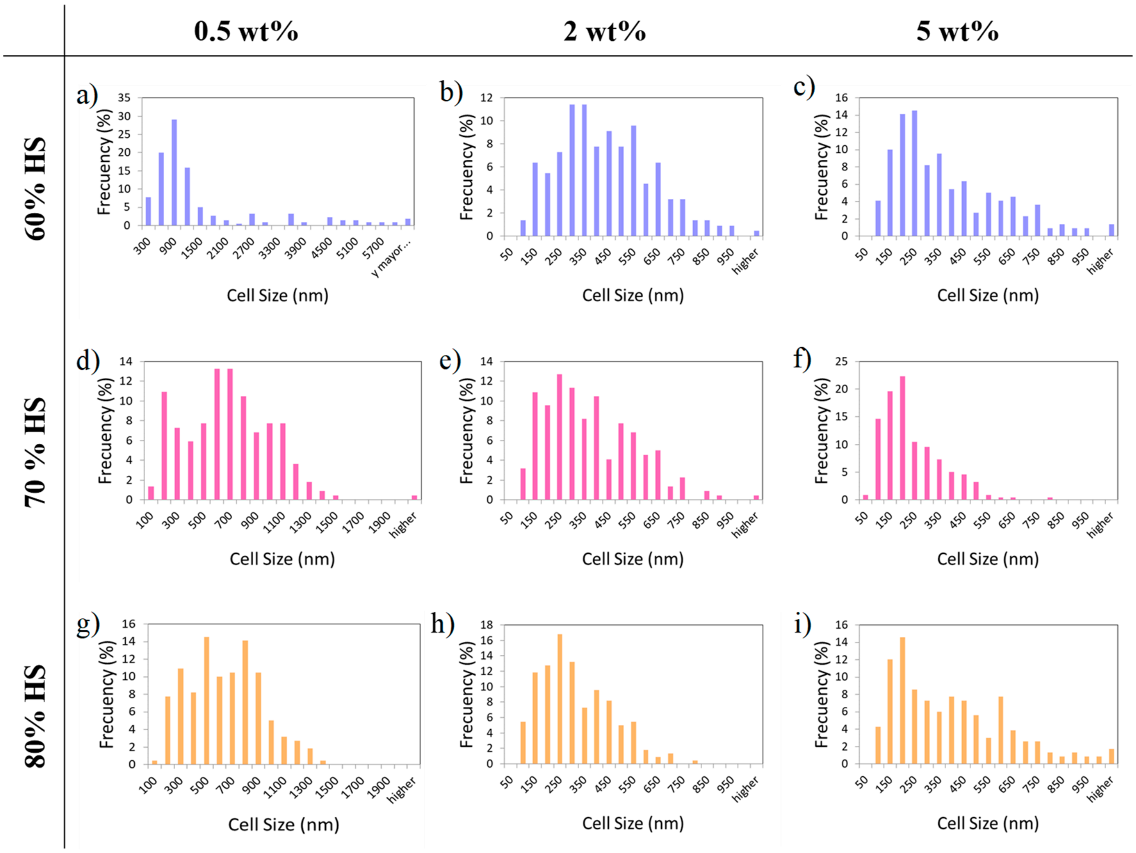


**Figure S9.** Core cell size distribution of the nanocellular region of the PMMA/TPU samples produced at normal conditions: (**a**) 0.5_60HS, (**b**) 2_60HS, (**c**) 5_60HS, (**d**) 0.5_70HS, (**e**) 2_70HS, (**f**) 5_70HS, (**g**) 0.5_80HS, (**h**) 2_80HS and (**i**) 5_80HS.

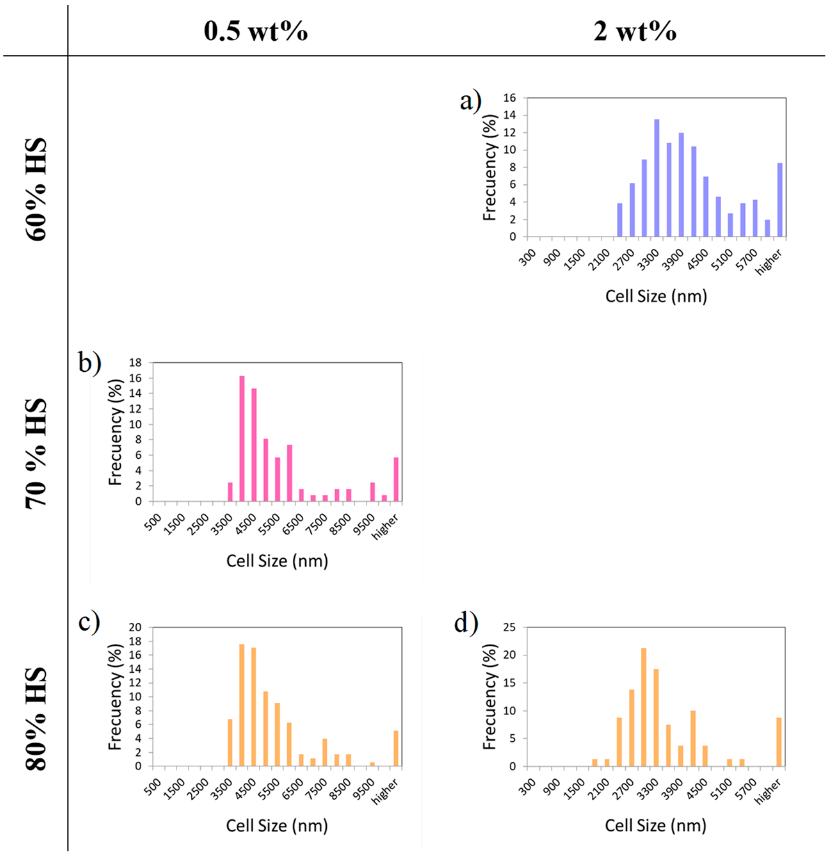


**Figure S10.** Core cell size distribution of the microcellular region of the PMMA/TPU samples produced at normal conditions: (**a**) 2_60HS, (**b**) 0.5_70HS, (**c**) 0.5_80HS, (**d**) 2_80HS.

## References

1. Guo, H.; Kumar, V. Solid-state poly(methyl methacrylate) (PMMA) nanofoams. Part I: Low-temperature CO2 sorption, diffusion, and the depression in PMMA glass transition. *Polymer (Guildf)* **2015**, *57*, 157–163, doi:10.1016/j.polymer.2014.12.029.
